# Arctigenin Exerts Neuroprotective Effect by Ameliorating Cortical Activities in Experimental Autoimmune Encephalomyelitis *In Vivo*

**DOI:** 10.3389/fimmu.2021.691590

**Published:** 2021-07-19

**Authors:** Liangpeng Wei, Zhenyi Xue, Baihui Lan, Shiyang Yuan, Yuanyuan Li, Cunle Guo, Rongxin Zhang, Ran Ding, Hui Shen

**Affiliations:** ^1^ School of Biomedical Engineering and Technology, Tianjin Medical University, Tianjin, China; ^2^ Department of Immunology, Key Laboratory of Immune Microenvironment and Diseases of Educational Ministry of China, Tianjin Key Laboratory of Cellular and Molecular Immunology, Tianjin Medical University, Tianjin, China; ^3^ Innovation Institute of Chinese Medicine, Shandong University of Traditional Chinese Medicine, Jinan, China; ^4^ Guangdong Province Key Laboratory for Biotechnology Drug Candidates, School of Life Sciences and Biopharmaceutics, Guangdong Pharmaceutical University, Guangzhou, China; ^5^ Academy for Advanced Interdisciplinary Studies, Peking University, Beijing, China; ^6^ Chinese Institute for Brain Research, Beijing, China; ^7^ Research Institute of Neurology, General Hospital, Tianjin Medical University, Tianjin, China

**Keywords:** EAE, arctigenin, two-photon Ca^2+^ imaging, *in vivo*, cortical activity, AMPA receptor

## Abstract

Multiple sclerosis (MS) is a chronic disease in the central nervous system (CNS), characterized by inflammatory cells that invade into the brain and the spinal cord. Among a bulk of different MS models, the most widely used and best understood rodent model is experimental autoimmune encephalomyelitis (EAE). Arctigenin, a botanical extract from Arctium lappa, is reported to exhibit pharmacological properties, including anti-inflammation and neuroprotection. However, the effects of arctigenin on neural activity attacked by inflammation in MS are still unclear. Here, we use two-photon calcium imaging to observe the activity of somatosensory cortex neurons in awake EAE mice *in vivo* and found added hyperactive cells, calcium influx, network connectivity, and synchronization, mainly at preclinical stage of EAE model. Besides, more silent cells and decreased calcium influx and reduced network synchronization accompanied by a compensatory rise in functional connectivity are found at the remission stage. Arctigenin treatment not only restricts inordinate individually neural spiking, calcium influx, and network activity at preclinical stage but also restores neuronal activity and communication at remission stage. In addition, we confirm that the frequency of AMPA receptor-mediated spontaneous excitatory postsynaptic current (sEPSC) is also increased at preclinical stage and can be blunted by arctigenin. These findings suggest that excitotoxicity characterized by calcium influx is involved in EAE at preclinical stage. What is more, arctigenin exerts neuroprotective effect by limiting hyperactivity at preclinical stage and ameliorates EAE symptoms, indicating that arctigenin could be a potential therapeutic drug for neuroprotection in MS-related neuropsychological disorders.

## Introduction

Multiple sclerosis (MS) is a chronic disease in which inflammatory processes attack the central nervous system (CNS) characterized by widespread inflammation, demyelination, gliosis, and neuropsychological disorders, resulting a huge healthcare burden (1, 2). In classical theory, MS has been hallmarked by demyelinating lesions in the focal white matter till now (3). However, with more investigation of MS, it is clear that detectable pathological changes have occurred in normally appearing white matter, as well as in the CNS grey matter because of the presence of focal grey matter lesions and gray matter atrophy (4). The analysis of functional connectivity obtained through resting-state functional magnetic resonance imaging (RS-fMRI) suggests a complex pattern of abnormal connection between the somatosensory network regions (5, 6). Recent reports have demonstrated that patients and animals with MS suffer from cognitive impairment and neuropsychological dysfunction in preclinical stage (7–9), indicating the destructive effects of inflammation on neurons and synapses in the earliest phases of MS (10).

As the most widely used model in MS research, experimental autoimmune encephalomyelitis (EAE) is usually accompanied by perivascular infiltration of leukocytes, demyelination, and axonal damage in cerebral cortex and white matter (11). Relapsing–remitting EAE (RR EAE) mouse models are suitable to imitate related aspects of relapsing–remitting MS (RRMS) (12). In addition, it is reported that EAE leads cortical layer five neuron loss and atrophy of the whole cerebral cortex, which strongly correlates with axonal damage (13–15). Besides, neural apoptosis and abnormal activity are also found in layer 2/3 cortex (11, 16).

Arctigenin isolated from an herbal medicine, Arctium, lappa, acts as potent bioactive component, which was widely used in Asian countries, especially in Korea (17, 18). Arctigenin has been reported to exhibit several pharmacological properties, including antidepressant (19, 20), anti-*T. gondii* (21), anti-inflammatory (22), and neuroprotective effects (23). Arctigenin can protect brain from ischemic stroke through inhibiting NLRP3 inflammasome activation (24). Additionally, arctigenin administration can protect dopaminergic neurons by eliciting potent antioxidant and anti-inflammatory effects and improve behavior changes of Parkinson’s disease (25). Arctigenin treatment reduced hematoma, gene expressions of inflammatory (IL-6 and TNF-α) and apoptosis (Caspase-3 and Bcl-2), and accelerated wound closure after a stab wound injury, indicating arctigenin possesses neuroprotective effects on brain tissue through anti-inflammatory and anti-apoptotic actions. What is more, arctigenin is hopeful for treatment of acute hepatitis diseases because arctigenin could remarkably reduce the congestion and necroinflammation of livers by suppressing concanavalin A–induced T lymphocyte proliferations (26).

Recently, we have reported that arctigenin can suppress the differentiation and proliferation of Th17 cells and ameliorate mice EAE symptoms (27). In our previous work, we have confirmed immunosuppressive properties of arctigenin because we found decreased Th1 and Th17 cells in the spleens and lymph node of arctigenin-treated mice by flow cytometry, as well as the expression of cytokines. What is more, histochemical assays of spinal cord sections indicated that arctigenin treatment reduced inflammation and demyelination in the central nervous system (CNS). However, the effects of arctigenin on the activities of cortical neurons attacked by inflammation in EAE animals *in vivo* are still unclear. Here, we used two-photon Ca2+ imaging to observe cortical activity in the somatosensory layer 2/3 cortex in living awake mice of EAE, and detected hyperactivity phenomenon exclusively at preclinical stage, including more hyperactive neurons, excessive calcium influx into cells, increased circuit functional connectivity and synchronization. Besides, we employed whole-cell patch-clamp recordings in acute brain cortex slices to record AMPA receptor-mediated sEPSC in preclinic phase, and the increased frequency of sEPSC was consistent with hyperactivity *in vivo*. Furthermore, increased fraction of silent neurons, reduced calcium influx, and network synchronization were found at the remission stage, indicating impaired neuronal activity at the third stage of disease. Meanwhile, cortical functional connectivity stayed at a high level as a compensation. Administration of arctigenin dramatically limited preclinically cortical hyperactivity and reversed the frequency of AMPAR-sEPSC to control level. Moreover, at remission stage, arctigenin decreased the number of silent neurons, restored neuronal activity and synchronization, and inhibited maladaptive functional connectivity. These results suggest that arctigenin exerts neuronal protection effects in EAE through attenuating glutamate-induced neurotoxicity at early time.

## Materials and Methods

### Animals

We purchased 8- to 10-week-old female C57BL/6 mice from the institute of zoology, Chinese Academy of Sciences. All animals used were maintained under a 12-h light/dark cycle and given enough food and water throughout the experiment and were housed in cages in the Tianjin medical university of China-approved animal facility. All experiments were supported by the Animal Care and Use Committee of Tianjin Medical University, in accordance with National Institutes of Health Guide for the Care and Use of Laboratory Animals.

### Stereotaxic Virus Injection

Mice were anesthetized with isoflurane (1–1.5%) and intraperitoneally injected with analgesic buprenorphine (0.3 mg/kg). The glass pipettes used to perform virus injection were beveled at 45° with a 10- to 15-µm opening. We inserted a suitable micro sample syringe controlled by a hydraulic manipulator (Narashige, MO10) into the pipette and backfilled the system with mineral oil when we loaded or infused the viral solution. To avoid virus leakage before arriving at the injection site, we filled the tip of glass pipette with ~1 nl saline before virus injection. After infusing solution, we kept the pipette in the brain for about 10 min and then withdrawn the syringe (~1 nl in volume) before pulling up the pipette to avoid backflow during withdrawal. About 30 nl volume of rAAV-hsyn-GCaMp6f-WPRE-hGH-containing solution (~ 2 × 10^13^ infectious units/ml) was injected into somatosensory cortex slowly (AP: ~ −1.5 mm, ML: ~ 2.0 mm, DV: ~ 0.35 mm) for imaging calcium activity with GCaMP6f.

### Craniotomy Surgery

Two weeks after virus injection, mice were anesthetized with isoflurane (1–1.5%) and injected with the analgesic buprenorphine (0.3 mg/kg). The skin on top of head was removed after local lidocaine injection (2%), and we performed a round craniotomy (~4 mm diameter) over the somatosensory cortex carefully and intermittently, using a cranial drill possessing a precisely machined tip steel burr with 0.5 mm in diameter. For better imaging results, the dura mater was removed by a forcep, with care taken to avoid damage to the cortex surface and blood vessels. A round glass coverslip (5mm in diameter) was sticked over the opening with biological glue. We used dental cement and cyanoacrylic glue (UHU) to fix the custom-made chamber onto the skull. After dental cement was set, mice were put back to cage and intraperitoneally injected with cephalosporin for 3 to 5 days to prevent infection at the craniotomy window. After craniotomy surgery, mice were allowed to recovery for 1 week.

### Induction and Treatment of EAE

To induce stable EAE model with a high success rate, we injected female mice (C57BL/6, aged 8–10 weeks) with myelin oligodendrocyte glycoprotein (MOG residues35–55). The sequence of peptide was Met-Glu-Val-Gly-Trp-Tyr-Arg-Ser-Pro-Phe-Ser-Arg-Val-Val-His-Leu-Tyr-Arg-Asn-Gly-Lys, and the peptide purity was more than 95% (CL. Bio-Scientific Co., LTD., Xi’an, China). We admixed 100 μg MOG35–55 peptide and complete Freund’s adjuvant containing 5 mg/ml of heat-killed H37Ra, a *Mycobacterium tuberculosis* strain (Difco Laboratories). And then, the mixture was injected into mice subcutaneously. Pertussis toxin (400 ng) (List Biological Laboratories) in PBS and NaCl (50 mM) was administered i.p. subsequently and the second time after 24 h (27). In animal models of EAE, mice usually exhibit disease symptoms within 7 to 14 days post induction and start to get alleviated about 20 days post induction. When EAE mice started to exhibit clinical symptoms, the tail was flaccid and drooping during walking. Then the clinical symptoms began getting worse. When the symptoms reached the worst, mice exhibited complete hindlimb and partial forelimb paralysis even death. Afterward, the mice that survived underwent alleviated in limb paralysis. The precise classification of different EAE stages was based on the actually clinical symptoms in our experiment. Because of the stability of EAE induction with MOG residues 35–55, the eight mice we induced for calcium imaging all showed clinical symptoms. Mice were randomly divided into two groups, one group was treated with arctigenin. For the intervention of EAE, arctigenin was injected (10 mg/kg, i.p.) daily from the first day after EAE induction, and DMSO was administrated as a vehicle treatment. We purchased arctigenin (purity > 98%) from Tianjin Shilan Technology company. To assess the disease symptoms, we used the below standard rating scale: 0, no obvious changes in motor functions; 1.0, limp tail; 2.0, limp tail and wobbly gait; 3.0, bilateral hind limb paralysis; 4.0, complete hindlimb and partial forelimb paralysis; and 5.0, death.

### Two-Photon Ca^2+^ Imaging *In Vivo*

A commercial Nikon A1R two-photon microscope system was used to perform two-photon calcium imaging. Two-photon excitation beam was emitted by a mode-locked Ti: Sa laser (model “Mai-Tai Deep See”, Spectra Physics). We utilized a water-immersion objective (Nikon) with 25 ×/1.10NA to perform imaging. The excitation wavelength was set to 910 nm for Gcamp6f calcium imaging experiments. For somatic imaging, the dimension of field-of-view (FOV) was set 200 µm × 200 µm. We acquired images of 512 × 512 pixels at 30-Hz frame rate. The average power reaching the cortical surface ranged from 30 to 40 mW, depending on the expression efficiency of virus and depth of imaging. Within an imaging time window of ~3 min (~1 min per time, three times in total) for each imaging, no sign of photo-damage was observed. Before the experiment, the mice were fixed under microscope objective frequently with the chamber to adapt to the imaging state for real calcium activity.

### Electrophysiological Recording

Separate seven mice underwent EAE induction for electrophysiological recording. We randomly chose three mice to inject arctigenin (10 mg/kg, i.p.) daily after induction. Day 7 to day 9 after induction, the mice that were under deep anesthetization by breathed isoflurane were decapitated. The brains were rapidly dissected in precooled, oxygenated standard artificial cerebrospinal fluid (ACSF) (120 mM NaCl, 2.5 mM KCl, 2.4, 1.25 mM NaH_2_PO_4_, 26 mM NaHCO_3_, 2 mM MgSO_4_, 2 mM CaCl_2_, 10 mM d-glucose; pH: 7.4). Both hemispheres were sliced coronally (350 µm) in cold ACSF. The slices containing somatosensory cortex were quickly transferred to 32°C ACSF for 30 min and then incubated at room temperature for at least 1 h. The slice was fixed in a recording chamber that was set on the fixed-stage of an upright Olympus BX50WI microscope (Olympus). The oxygenated ACSF was perfused through recording chamber continuously. Pyramidal neurons in layer 2/3 were optically identified and whole-cell patched for recording. Picrotoxin (50 µM), D-AP5 (20 µM), and TTX (1 µM) were applied to pharmacologically isolate the AMPA receptor-mediated sEPSCs, with voltage clamp-mode at −70 mV. The resistance of glass pipettes for recording were 5 to 7 MΩ (intracellular solution containing: 130 mM CsMeSO_3_, 8 mM NaCl, 10 mM HEPES, 0.3 mM Na-GTP, 4 mM Mg-ATP, 5 mM QX314, 0.2 mM EGTA; pH: 7.3). An Axon 700B amplifier (AXON Instrument) was utilized to record the electrical signals. The data were digitized at 20 kHz and filtered at 10 kHz by using a Digidata-1440B system with pclamp 10.1 software (Molecular Device).

### Data Analysis

The calcium imaging data were calibrated for motion artifacts and analyzed with custom-written software in Matlab 8 (Mathworks). We visually identified and drawn regions of interests (ROIs) based on fluorescence intensity to acquire fluorescence signals from imaging data. The Ca^2+^ signals were represented by relative fluorescence changes calculated as Δf/f = (f − f0)/f0. In which f was estimated by averaging fluorescence of all pixels within each specified ROI, and f0 represented the baseline fluorescence of ROI estimated as the 25th percentile of the fluorescence within a sliding time window. Cortical functional connectivity is defined as a strong temporal correspondence of events between two neurons (28). Drastic motion imaging data were excluded from the analyses. The Kolmogorov–Smirnov (K-S) test was used to determine normality of all data sets. Statistical significance was evaluated using Two-sample Mann-Whitney test or ANOVA with Kruskal-Wallis test. Distribution histogram and cumulative distribution were used to compare distribution difference of groups in different periods of EAE. Further experimental results were represented as mean ± SEM, P < 0.05 was regarded as statistically significant.

## Results

### Two-Photon Ca^2+^ Imaging in Layer 2/3 of Somatosensory Cortex *In Vivo* in Living Awake Mice

**Figure 1:**
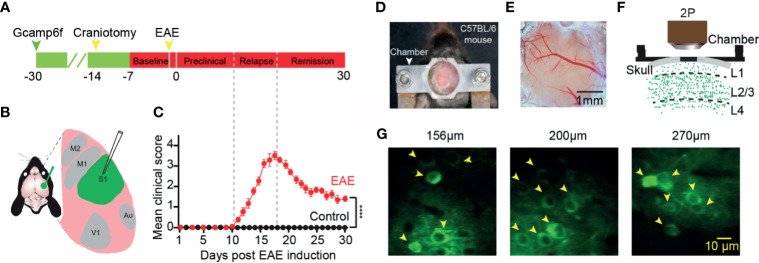
Imaging neuronal activity in somatosensory cortex in awake mice *in vivo*. **(A)** Timeline for two photon imaging of neuronal activity *in vivo*. Green arrow, indicates virus injection time point; Craniotomy was engaged for chronic imaging; Green line, preparation time for virus expression (about three weeks); Red line represents two-photon imaging time course (about 1 week of pre-imaging for adaption and 1 month after EAE induction). **(B)** Illustration of virus injection sites. **(C)** Mean daily clinical score of mice recorded from day 1 to day 30 after EAE induction, data shown as mean ± SEM, n=6 mice per group, ****p < 0.0001, Two-sample Mann-Whitney test. **(D)** Chamber fixed to the cranium of the mouse for chronic two-photon imaging. **(E)** Bright field image of a cranial window at somatosensory cortex. **(F)** Schematic of two photon imaging process. **(G)** Individual *in vivo* two photon images at different cortical depths in layer 2/3 (left, 156µm; middle, 200µm; right, 270µm).

We first injected an ultra-sensitive genetically encoded calcium indicator, GCaMP6f (29), into the layer 2/3 of somatosensory cortex (**Figures 1A, B**). A chronic cranial window on cortex was established to observe neuronal activity in the awake mouse *in vivo* for at least one month (**Figures 1D–F**). Healthily control wild-type C57BL/6 mice exhibited spontaneous neuronal activity at different cortical depths (**Figure 1G**). We induced EAE with myelin oligodendrocyte glycoprotein (MOG residues 35–55) injected into the mice *via* subcutaneous injection (27). The values of the disease clinical score were recorded every day after EAE induction. According to the clinical scores, we classified the process of the disease to three stages: “preclinical”, “relapse”, and “remission” stage (**Figures 1A, C**). In the preclinical stage, there is no clinical symptom in EAE mice, whereas in the relapse and remission stage, the obvious symptom came up and lasted several weeks, and the healthy wild-type mice did not exhibit any clear clinical symptoms.

### Arctigenin Relieves EAE Symptoms

**Figure 2:**
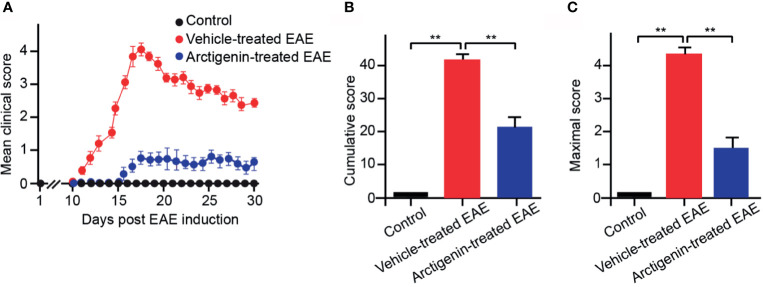
Arctigenin relieves the symptoms of EAE. **(A)** Arctigenin was intraperitoneally injected everyday (10 mg/kg) at the beginning after EAE induction. The mean of the daily clinical scores recorded from day 1 to day 30 post-induction after treatment with 10 mg/kg Arctigenin. **(B)** The mean of the total of the daily clinical scores observed between day 1 and day 30. **(C)** The maximum clinical score of individual mice between day 1 and day 30. All data are expressed as mean ± SEM, n=6 mice per group, **p < 0.01, one-way ANOVA with Kruskal-Wallis test.

Arctigenin was injected intraperitoneally every day (10 mg/kg) from the beginning after EAE induction to determine its effects on EAE. The arctigenin-treated mice had a delayed onset of clinical symptoms, approximately 5 days (**Figure 2A**) compared with the vehicle-treated EAE mice. Besides, the arctigenin-treated group had evidently reduced clinical scores throughout the disease. The severity of disease was assessed by cumulative and maximum clinical scores (**Figures 2B, C**). EAE mice without arctigenin treatment had significantly higher cumulative clinical score (40.55 ± 6.42) than arctigenin-treated mice (22.00 ± 4.07) (**Figure 2B**). These results suggest that arctigenin relieves EAE mice’s clinical symptoms.

### Emergence of More Hyperactive Neurons in Early EAE

**Figure 3:**
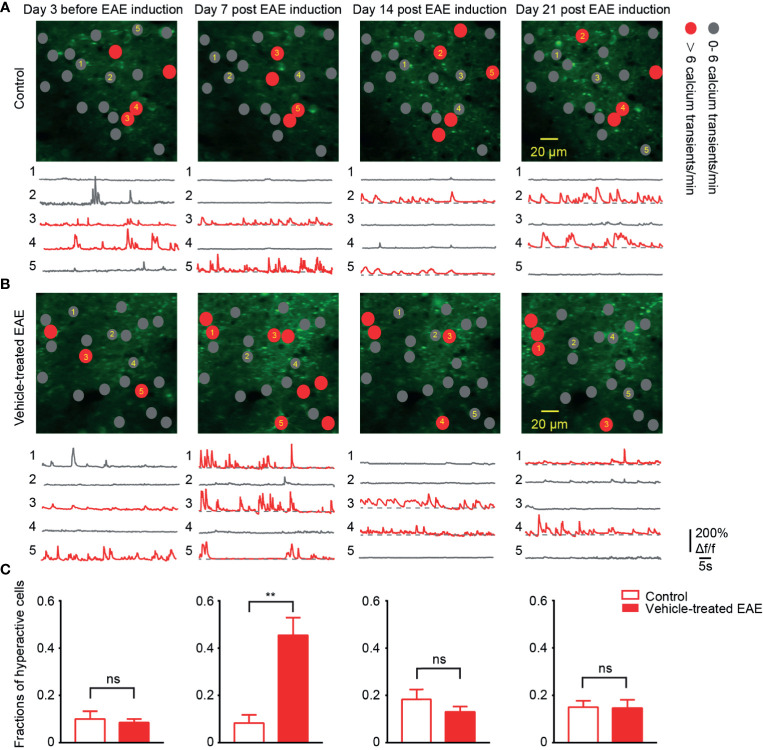
More hyperactive neurons in somatosensory cortex in preclinical stage of EAE. **(A, B)** Top, representative images and activity maps in layer 2/3 cortical neurons in 3 days before and 7, 14, 21 days after EAE induction (from left to right) in control **(A)** and vehicle-treated EAE mice **(B)**. Gray solid circle means 0- 6 Ca^2+^ transients/min and red solid circle means > 6 Ca^2+^ transients/min (hyperactive cells); Bottom, calcium transients of the marked neurons. **(C)** Quantitative data of fraction of hyperactive cells in imaged regions for **(A, B)** on the 3 days before induction (n=9 images from three mice in control group and n=11 images from four mice in vehicle-treated EAE group), the 7th day (n=9 images from three mice in control group and n=20 images from four mice in vehicle-treated EAE group), the 14th day (n=9 images from three mice in control group and n=11 images from four mice in vehicle-treated EAE group) and the 21st day (n=9 images from three mice in control group and n=10 images from four mice in vehicle-treated EAE group) after induction. Data are shown as mean ± SEM., **P < 0.01, ns, not significant, Two-sample Kolmogorov-Smirnov test.

To chronically monitor neurons activity in layer 2/3 of somatosensory cortex, we performed two-photon calcium imaging at 8:00 a.m. daily for about 40 days. Representative calcium images of the same region and activity traces of neurons at four time points in different periods of the experiment are shown (**Figures 3A, B**). In healthy control mice, around 12% of neural population exhibited hyperactivity (>6 transients/min) throughout entire chronic recordings. Moreover, in EAE mice, the fraction of hyperactive cells also stayed at the same level with control group both in the relapse (13%) and remission stage (14%). However, it is noteworthy that, on the 7th day post EAE induction, we observed a significant increase (up to 40%) in the mean fraction of hyperactive neurons (**Figure 3C**). This extraordinary proportion of hyperactive neurons indicates the change of cortical activity pattern in early EAE.

### Arctigenin Restricts the Increase in Fraction of Hyperactive at Preclinical Stage and Reduced Silent Neurons at Remission Stage of EAE *In Vivo*

**Figure 4:**
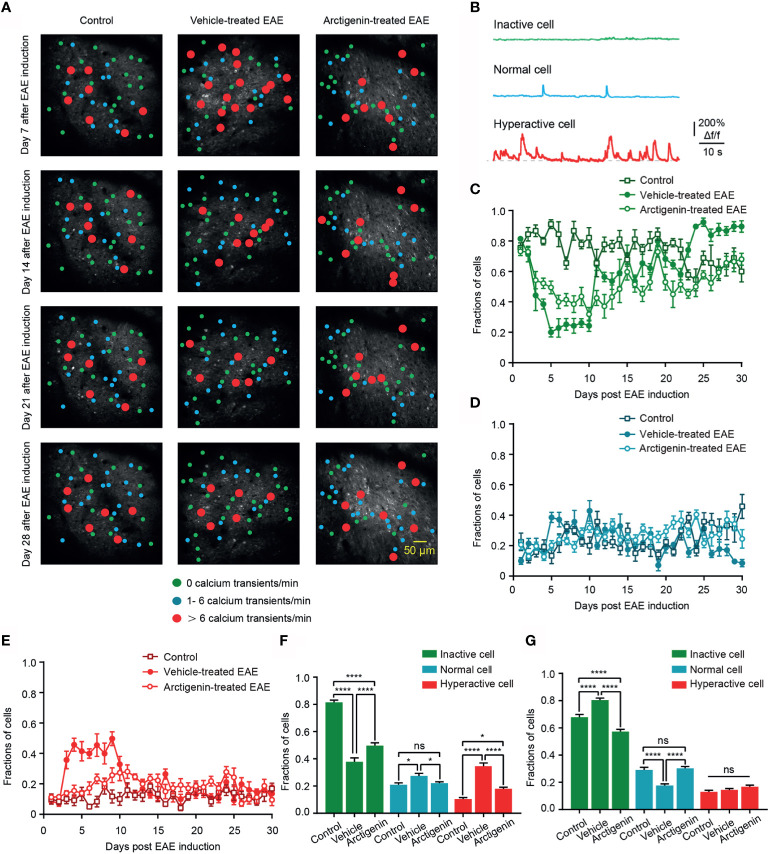
Arctigenin limits the increase in proportion of hyperactive neurons at preclinical stage and inactive neurons at remission stage of EAE *in vivo*. **(A)** Representative image and activity map in layer 2/3 cortical neurons on day 7, day 14, day 21 and day 28 after EAE induction in control (left), vehicle-treated (middle) and Arctigenin-treated mice (right). **(B)** Neurons are categorized into three types based on the frequency of spontaneous calcium transients. Inactive cells are depicted in green (no Ca^2+^ transient), normal in blue ( 1- 6 Ca^2+^ transients/min), and hyperactive in red ( >  6 Ca^2+^ transients/min). **(C–E)** Daily fractions of inactive cells **(C)**, normal cells **(D)** and hyperactive cells **(E)** in imaged regions during chronic recording, n=180 images in three control mice, n=210 images in four vehicle-treated EAE mice, n=210 images in four arctigenin-treated EAE mice. **(F)** Integrated fraction of three category cells in control (n=60 images in three mice), vehicle-treated (n=70 images in four mice) and Arctigenin-treated (n=70 images in four mice) group at preclinic stage, *P < 0.05, ****P < 0.0001, ns, not significant, one-way ANOVA with Kruskal-Wallis test. **(G)** Integrated fraction of three category cells in control (n=72 images in three mice), vehicle-treated (n=84 images in four mice) and Arctigenin-treated (n=84 images in four mice) group at remission stage, ****P < 0.0001, ns, not significant, one-way ANOVA with Kruskal-Wallis test.

Based on the calcium transients, cells were further classified to three categories (30), inactive cell (no Ca^2+^ transient in recording time), normal cell (1– 6 Ca^2+^ transients/min), and hyperactive cell (> 6 Ca^2+^ transients/min) to observe the details of cortical activity in EAE (**Figures 4A, B**). The distribution of hyperactive cell in activity map shows more hyperactive neurons in vehicle-treated EAE mice on the 7th day after induction, whereas the increase was not obvious in arctigenin-treated mice (**Figure 4A**). In addition, from the chronic recording of three kinds of cells, we found that the fraction of hyperactive cells abruptly increased as early as 3 days after EAE induction and remained at a high level for about a week. However, arctigenin treatment made this increase slow and mild (**Figure 4E**). Furthermore, the more inactive cells and less normally active cells appeared during late period in vehicle-treated EAE mice while arctigenin reversed these change (**Figures 4C, D**). Apart from that, we summarized the fraction of three category cells in the preclinical stage (day 1 to day 10 after induction) and remission stage (day 19 to day 30 after induction) (**Figures 4F, G**). Notably, the percentage of hyperactive cells, as well as normal cells in vehicle-treated EAE group, was significantly increased compared with control mice at preclinical stage and arctigenin reversed the increase to a considerable degree (**Figure 4F**). Meanwhile, at the remission stage of EAE, the fraction of inactive cells was obviously increased in vehicle-treated mice, and the normally active cells were reduced significantly, which means cortical silence during the late period of disease. However, arctigenin compromised these effects of EAE induction (**Figure 4G**). Taken together, these results demonstrate that arctigenin limited the growing in numbers of cortical hyperactive neurons at preclinic stage of EAE. Besides, arctigenin treatment reduced the number of silent cells and facilitated normal activity of cells at remission stage.

### Arctigenin Limits a Surge in Calcium Transient Amplitude of Cortical Neurons at Preclinical EAE and Reversed Calcium Influx at Remission Stage

**Figure 5:**
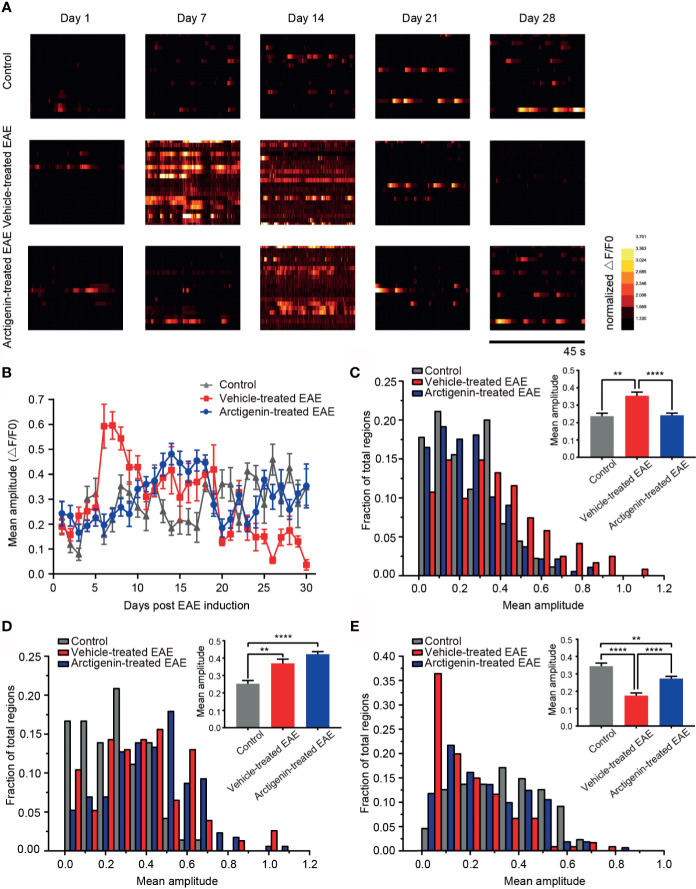
Arctigenin modulated the change of calcium transient amplitude induced by EAE. **(A)** Heat maps depicting changes in the calcium transient amplitude (ΔF/F0) for a representative neuronal population at different time point in control mice, vehicle-treated EAE mice and arctigenin-treated EAE mice. Each row represents a single cell. The amplitude of each cell is normalized to the minimum value of its own calcium transient. **(B)** Mean calcium transient amplitude of imaged regions daily after EAE induction. **(C–E)** Cumulative distribution histogram and statistical histogram (inset) of calcium transient amplitudes for integration of three stages respectively, including preclinical stage (**C**, n=99 images in three control mice, n=129 images in four vehicle-treated EAE mice, n=160 images in five arctigenin-treated EAE mice), relapse stage (**D**, n=72 images in three control mice, n=77 images in four vehicle-treated EAE mice, n=206 images in five arctigenin-treated EAE mice) and remission stage (**E**, n=99 images in three control mice, n=126 images in four vehicle-treated EAE mice, n=245 images in five arctigenin-treated EAE mice), **p < 0.01, ****p < 0.0001, one-way ANOVA with Kruskal-Wallis test.

To characterize the change of intracellular calcium concentration during EAE and arctigenin-treated process, we analyzed the mean amplitude of calcium transient in regions of view. Consistent with previous results, on the 7th day post EAE induction, the calcium transient amplitude increased significantly in vehicle-treated EAE mice compared with arctigenin-treated group. Moreover, it is quite unexpected that there were enhancements of calcium transient amplitude in control mice and arctigenin-treated group, whereas there was an obvious decrease in vehicle-treated mice on the 28th day (**Figure 5A**). From the day 6 after induction, the amplitude in vehicle- treated EAE group suddenly increased and gradually dropped to the original level, then further decreased to a very low level at late EAE stage. However, the increase of calcium transient amplitude in arctigenin-treated mice was slow and moderate in early EAE, like the increase of hyperactive cells described above. What is more, the amplitude of control and arctigenin-treated mice experienced a slight enhancement in the late period. This enhancement may be due to chronic and repeated touching mice to operate two-photon imaging (**Figure 5B**). In addition, we summarized the amplitude of three groups in preclinical, relapse, and remission stage, respectively. And we found that the amplitude in vehicle-treated mice was significantly increased at the preclinical stage, whereas arctigenin administration inhibited this preclinical increase (**Figure 5C**). However, at the relapse stage, the amplitude began to grow in arctigenin-treated mice and exceeded vehicle-treated EAE mice (**Figure 5D**). Besides, the amplitude of calcium transient in vehicle-treated group significantly decreased at remission stage but arctigenin reversed this change to some extent (**Figure 5E**). Collectively, these results indicate that more calcium influx accompanied with neuronal activity at preclinic EAE stage resulted in functional deficits of cortical cells at remission stage. However, arctigenin exerted neuronal protection effects through delaying and attenuating the abnormal calcium influx at preclinical stage, indeed decreased silent cells caused by EAE induction.

### Restoration of Normal Functional Connectivity and Phase Synchronization of Cortical Network at Preclinical and Remission Stage by Arctigenin Administration

**Figure 6:**
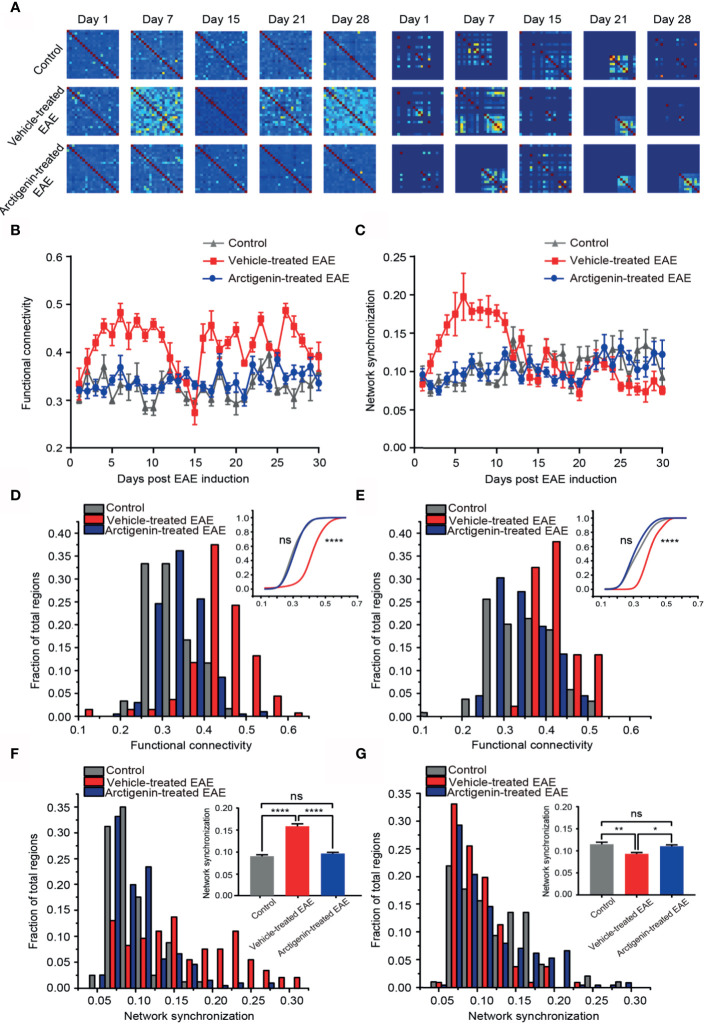
Restoration of normal functional connectivity and synchronization of cortical network by Arctigenin administration in EAE mice. **(A)** Representative correlation matrices measuring network functional connectivity (left) and phase synchronization between each cell and every other cell over time during process of EAE and Arctigenin treatment. **(B)** The value of functional connectivity for daily observation. **(C)** The value of network synchronization for daily observation. **(D)** Distribution histogram and cumulative distribution (inset) of integrated cortical functional connectivity at preclinical stage, n=60 images in three control mice, n=129 images in four vehicle-treated EAE mice, n=187 images in five Arctigenin treated EAE mice, control *vs* Arctigenin treated EAE, ns, not significant; Arctigenin treated EAE *vs* vehicle-treated EAE, ****p < 0.0001, one-way ANOVA with Kruskal-Wallis test. **(E)** Distribution histogram and cumulative distribution of integrated cortical functional connectivity at remission stage, n=66 images in three control mice, n=100 images in four vehicle-treated EAE mice, n=245 images in five arctigenin-treated EAE mice, control *vs* Arctigenin treated EAE, ns, not significant; arctigenin-treated EAE *vs* vehicle-treated EAE, ****p < 0.0001, one-way ANOVA with Kruskal-Wallis test. **(F)** Distribution histogram and statistical histogram (inset) of integrated network synchronization at preclinical stage, n=80 images in three control mice, n=146 images in four vehicle-treated EAE mice, n=196 images in five arctigenin treated EAE mice, ****p < 0.0001, ns, not significant, one-way ANOVA with Kruskal-Wallis test. **(G)** Distribution histogram and statistical histogram of integrated network synchronization at permission stage, n=96 images in three control mice, n=106 images in four vehicle-treated EAE mice, n=226 images in five arctigenin treated EAE mice, **p < 0.01, *p < 0.05, ns, not significant, one-way ANOVA with Kruskal-Wallis test.

Functional connectivity is defined as a key indicator of temporal correspondence of calcium transients between two individual neurons. It represents the temporal causality of calcium events among neurons, which means communication in function. Synchrony is the temporal consistence of distributed neuronal activity, which serves as the ability of transferring information among neurons. The high synchrony means more calcium events occur at the same time point. We used these two parameters to estimate temporal network activity patterns through calculating correlation and synchrony matrices quantifying network functional connectivity (**Figure 6A**, left) and synchronization (**Figure 6A**, right) among neurons in imaged regions, respectively. One week after EAE induction, both functional connectivity and synchronization of vehicle-treated EAE mice were extremely higher in comparison to arctigenin-treated group (**Figures 6A–C**). Indeed, at the entire preclinical stage, the cortical functional connectivity and synchronization in vehicle-treated mice increased starkly, and arctigenin inhibited the abnormal increase (**Figures 6D, F**). However, after a long duration of steady enhancement, the functional connectivity continued to decrease and reached to the lowest level on the day 15 post induction in vehicle-treated EAE mice (**Figure 6B**). It is worthy to note that from the end of relapse stage, the network connectivity was precipitously elevated and maintained a high level throughout entire remission stage in vehicle-treated group (**Figure 6E**). Intriguingly, the network synchronization was remained a low level at remission stage in vehicle-treated mice, indicating deficits in information transfer (**Figure 6G**). However, arctigenin rescued these abnormal effects of EAE induction. In conjunction with the data above, these results suggest the cortical hyperactive microcircuit activity at preclinical stage of EAE, whereas arctigenin significantly prevented network hyperactivity and reversed maladaptive functional connectivity and decreased synchrony upon recovery at remission stage.

### Arctigenin Blunts Increased Frequency of AMPA Receptor-Mediated sEPSCs in Preclinic EAE

**Figure 7:**
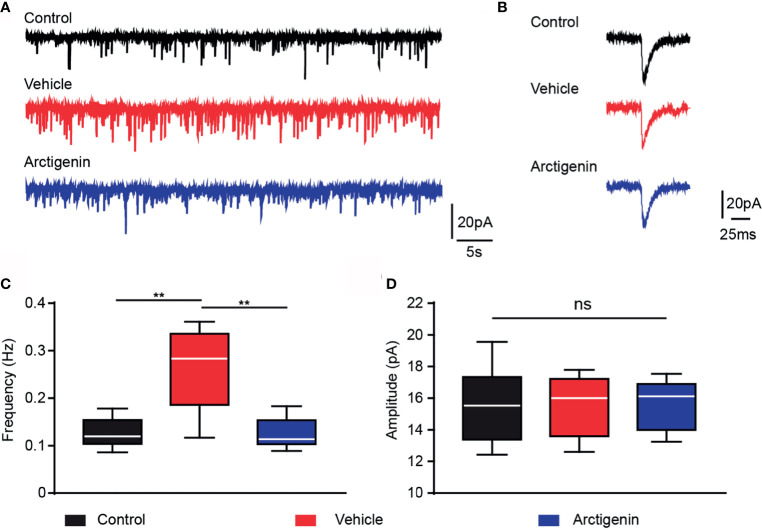
Arctigenin modulates AMPA receptor-mediated sEPSCs. Whole-cell recordings were performed in voltage-clamp mode in acute cortex slices of control and preclinical stage mice. **(A)** Representative current traces of pharmacologically isolated AMPA-receptor-mediated sEPSCs in the three groups (black, control group; red, vehicle-treated group; blue, arctigenin-treated group). **(B)** Representative traces of individual sEPSC. **(C, D)** Quantification of sEPSC frequency **(C)** and amplitude **(D)**. Box-and-whisker plot indicates the median value (center line), the 25th to 75th percentile (box) and the 10th to 90th percentile (whiskers), n=10 neurons from four control mice, n=11 neurons from five vehicle-treated mice, n=8 neurons from four arctigenin-treated mice, **P < 0.01, ns, not significant, one-way ANOVA with Kruskal-Wallis test.

Activation of glutamate AMPA type receptors could trigger calcium influx. Excessively activation of AMPA receptors may result in glutamate excitotoxicity, which might contribute to the neuropsychological disorders in MS (31, 32). We hypothesize that arctigenin attenuates cortical hyperactivity, which may act through AMPA receptor-mediated synaptic transmission. We recorded AMPA sEPSCs of single neurons in acute cortical brain slices of healthy controls and EAE mice in preclinical stage (day 7 to day 9 after induction). The frequency of sEPSCs was significantly increased in cells of vehicle-treated EAE mice (0.26 ± 0.03 Hz) in comparison to control mice (0.12 ± 0.01 Hz), and the frequency of sEPSCs was significantly decreased to control level in cells of actigenin-treated EAE mice (0.12 ± 0.01 Hz) (**Figures 7A, C**). However, there is no difference in the mean amplitude of sEPSCs between the three groups (**Figures 7B, D**). The increased frequency of AMPA receptors mediated sEPSCs, but not amplitude, can be probably explained by a presynaptic enhancement of glutamate transmission, which is compatible with increased neuron activity indicated by Ca^2+^ transients in preclinic stage *in vivo*.

## Discussion

In our present study, with chronic real-time two-photon Ca^2+^ imaging in somatosensory cortex, we discovered engagement of cortical hyperactivity at preclinical stage of EAE, including increase in fraction of hyperactive cells, calcium influx into cells, cortical functional connectivity, and network synchronization. However, at preclinical stage, the mice did not exhibit related clinical symptoms, as measured by the clinical scores. In sharp contrast, EAE mice exhibited a bulk of inactive cells and a few normally active cells at remission stage. Parallel to this, the amplitude of calcium transient in EAE mice was very low in remission stage, which means defective neuronal activity. What is more, the decreased network synchrony at remission stage means poor efficiency of information transmission. As a compensation, the functional connectivity stayed at a high level at remission stage to reverse the dysregulation of network circuit as the disease progresses (16, 33). Arctigenin treatment can suppress the hyperactivity phenomenon to a considerable degree at preclinical stage while restoring neuronal activity and network function at remission stage. Indeed, arctigenin delayed the onset of clinical symptoms and ameliorated severity. Besides, we did whole-cell voltage clamp patch in acute cortex slices to record the glutamate AMPA receptors sEPSC and found increased frequency of AMPAR sEPSC in preclinic phase, which can be reversed to normal level by administration of arctigenin.

Inflammation response emerged in brain cortex during the early period of preclinical stage. In MOG33-35 residues induced EAE model, leukocyte rolling and adhesion is already apparent in the cerebral microvasculature at day 7 after induction (34). Similarly, the anti-inflammatory cytokines TGF-β and IL-10 in brain are detectable early in 100 µg MOG33-35–induced EAE, with production peaks on the day 7 (35). The release of IL-17 and IFN-γ on day 7 is observed and increased in CNS. TGF-β and IL-6 act concertedly to drive T-cell expansion and also differentiation from naïve to a Th17 phenotype (36), or act through activating astrocytes, microglia, and antigen presentation in the CNS (37, 38). Additionally, IL-11 is one of the cytokines, which are most highly increased in the cerebrospinal fluid from early RRMS patients, and also can lead to the differentiation and expansion of Th17 cells in MS during the earliest stages (39). These evidences support that early inflammation has occurred in the CNS of EAE mice at early stage, in which the Th17 cells are the key encephalitogenic population (40). Indeed, targeting Th17 cells will ameliorate MS-related neuropsychological disorders. For example, montelukast limits Th17 cells response by inhibiting the cysteinyl leukotriene receptor (CysLTR1) signaling and alleviates inflammation in EAE (41). Moreover, cornuside exerts anti-inflammatory and immunosuppressive effects and alleviates symptoms of EAE probably through inhibiting Th17 cells (42). Besides, overexpression of miRNA-467b could suppress Th17 cell differentiation and reduce pathologic changes (inflammation and demyelination) in CNS (43). Importantly, Th17 cells have been reported to form synapse-like contacts with neurons directly, and induce neuronal intracellular Ca^2+^ concentration to undergo serious and localized fluctuation, which could be partially reversed, as an early sign of neural damage (44). Here, we detected more cortical hyperactive neurons in preclinical stage of EAE. Moreover, the calcium transient amplitude was obviously elevated at the same time, which means more calcium influx into cells. Because higher concentration of glutamate increases calcium transient amplitude in excitotoxic situation (45), it is consistent with more proportion of hyperactive neurons we detected in the preclinical stage, which facilitates glutamate release and results in more glutamate in cortical extracellular space. Furthermore, the network functional connectivity and synchronization also increased immediately after EAE induction, which indicates dysregulation of cortical microcircuit activity. Of note, we observed the significant increase in fraction of hyperactive neurons from as early as the third day after induction. Combining calcium dynamics of single-cell and network circuit, we confirmed the cortical hyperactivity in preclinical stage of EAE, which may have resulted from early inflammation in CNS. However, recently, Erik Ellwardt et al. applied two-photon Ca^2+^ imaging to assess cortical microcircuits in neuroinflammation in MS. They found the hyperactive cells only in the remission stage not in the preclinical stage (16). We concluded that the difference between us may be due to the diversity of animal and induction drugs, because proteolipid protein (PLP) and MOG induce different EAE models and autoimmune responses (46). What is more, they used OGB-1 as calcium indicator which needs to be loaded to label neurons before imaging in anesthetized mice, while we repeatedly imaged the same region of cortex in awake mice.

Autoimmune-induced glutamatergic upregulation is involved in many disorders, such as Alzheimer (47) and MS (48). AMPA receptor activation induces calcium influx to participate in pathological pathways, playing an important role in excitotoxicity. In rats with minimal hepatic encephalopathy, peripheral inflammation induces altered hippocampal neurotransmission and impairs spatial learning and memory (49). It is confirmed that the frequency of AMPA receptor-mediated sEPSC is increased at preclinical stage in the striatum of EAE mice with altered GluA1 protein composition of postsynaptic density (PSD) (50). Moreover, in a previous study, mice with EAE showed increased AMPA receptor expression in hippocampus, and sildenafil was able to reduce excitotoxicity by decreasing expression of the receptors (51). Because activated immune cells release large quantities of glutamate, AMPA/kainite antagonists can reduce autoimmune demyelination and result in substantial amelioration (52). In our study, we found that there was an evident abnormality of glutamatergic synaptic transmission in cerebral cortex at EAE preclinical stage, considering the increased frequency of AMPA receptor sEPSC. This abnormal AMPA receptor-mediated transmission may account for the cortical hyperactivity.

Remission stage is an important and long stage in which patients are allowed to recover from severe clinical symptoms. Notably, we found obvious hypoactivity at EAE remission stage. More neurons became silent and the amplitude of calcium transient reduced to a very low level in vehicle-treated EAE mice, reflecting deficits in neuronal activity. Besides, the network synchronization also decreased significantly at remission stage, which means inefficiency information transfer between neurons. Meanwhile, circuit functional connectivity stayed at a maladaptively high level to compromise the hypoactivity, as functional reorganization in brain cortex. Since multiple lines of evidence support that there is a close correlation between excitotoxicity and neurodegeneration, we speculate that at preclinical stage, early inflammatory response caused excessive extracellular glutamate concentration. As a result, AMPA receptors were over activated then facilitated neuronal hyperactivity and abnormal calcium influx. Finally, the massive calcium influx induced excitotoxicity and resulted in neuronal activity deficits, poor information transfer, and maladaptive functional connectivity at remission stage. However, arctigenin restricted inflammation induced neuronal hyperactivity and excessive calcium influx at preclinical stage and protected neurons from excitotoxic injury and death (53, 54) throughout the disease, whereas arctigenin prevented this hypoactivity phenomenon and restored cortical functional connectivity to normal level at remission stage.

In summary, we confirmed that the protective effect of arctigenin on EAE mice mainly in aspects of neuronal activity and network pattern. We have shown that arctigenin can suppress neuronal hyperactivity and abnormal calcium influx by limiting inordinate glutamate synaptic transmission at EAE preclinical stage, perhaps through attenuating initial immune attack in CNS. Besides, arctigenin reversed insulted activity of individual neurons and network spike patterns at remission stage. These results suggest that arctigenin could be a potential therapeutic drug for MS-related neuropsychological disorders at early time.

## Data Availability Statement

The original contributions presented in the study are included in the article/supplementary material. Further inquiries can be directed to the corresponding authors.

## Ethics Statement

The animal study was reviewed and approved by Animal Care and Use Committee of Tianjin medical university.

## Author Contributions

LW performed partial two-photon imaging and electrophysiological experiments, wrote the manuscript. ZX performed EAE induction and partial data analysis. BL performed partial data analysis. SY performed partial two-photon imaging. YL performed partial data analysis. CG scored clinical symptoms of animals. RZ designed the experiments. RD designed the experiments and analyzed partial data. HS designed the experiments and wrote the manuscript. All authors contributed to the article and approved the submitted version.

## Funding

This work was supported by grants from the National Natural Science Foundation of China (grant numbers 62027812, 81771470) and the Natural Science Foundation of Tianjin, China (grant number 19JCQNJC11000).

## Conflict of Interest

The authors declare that the research was conducted in the absence of any commercial or financial relationships that could be construed as a potential conflict of interest.
